# Light-driven molecular switching achieves 6-order magnitude conductance change in OPE dimers

**DOI:** 10.1039/d5na00553a

**Published:** 2025-10-24

**Authors:** Asma Alajmi, Bashayer Alanazi, Karimah Alresheedi, Kholood Alharbi, William D. J. Tremlett, Nicholas J. Long, Colin Lambert, Ali Ismael

**Affiliations:** a Physics Department, Lancaster University Lancaster LA1 4YB UK c.lambert@lancaster.ac.uk k.ismael@lancaster.ac.uk; b Department of Physics, College of Science and Humanities in Al-Kharj, Prince Sattam bin Abdulaziz University Al-Kharj 11942 Saudi Arabia; c Department of Physics, College of Science, Northern Border University Arar Saudi Arabia; d Department of Physics, College of Science, Qassim University Buraydah 51402 Saudi Arabia; e Department of Chemistry, Imperial College London London W12 0BZ UK; f Department of Physics, College of Education for Pure Science, Tikrit University Tikrit Iraq

## Abstract

Functional molecular devices have garnered significant research interest over the past ten years due to their promising potential for applications in both non-volatile memory and novel computing architectures. In this work, we investigate light-induced switching of electrical conductance in linear oligo(phenylene-ethylene)-based (OPE) molecules that incorporate an azobenzene bridging unit to form OPE dimers. We demonstrate that a light stimulus can cause electron transport through the molecule to switch from constructive to destructive quantum interference through an *E* to *Z* (*trans* to *cis*) azobenzene isomerisation, leading to an on–off conductance ratio of up to 6 orders of magnitude, which is comparable with the best molecular switches obtained to date.

## Abstract 2

(a) OPE3 *meta* and *para*-junctions. (b) Transmission *T*(*E*) *vs.* energy.

## Graphical abstract

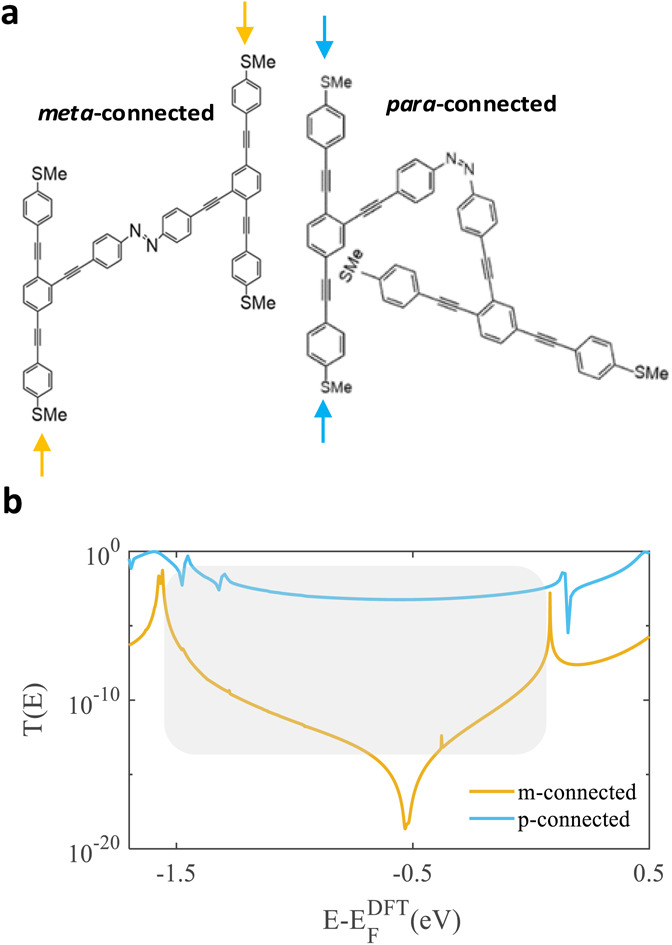

## Introduction

Knowledge about charge transport through discrete chemical compounds is crucial to enable the development of single-molecule electronic devices.^1,2^ In the past two decades, many molecular building blocks and assemblies have been studied as channel materials for single molecule device applications^3^ including organic conjugated molecules exhibiting room-temperature quantum interference (QI) effects.^4–7^ In a single-molecule junction (SMJ), the molecules that form the backbone of the junction are the primary mediator of electron transfer across the junction. Hence, the bond switching mechanism assumes that it is the change of these chemical bonds that can be the tool to tailor the transmission of the electrons through molecules. Charge transport through single molecules placed between two metallic electrodes is governed by several factors, such as the compositions of the anchor groups, the length of the molecule, the properties of the spacers, and the electronic structures of the aromatic subunits.^8–10^

Other critical properties are molecular conformation, the energy gap between the HOMO (Highest Occupied Molecular Orbital) and LUMO (Lowest Unoccupied Molecular Orbital),^11^ the positioning of this gap with respect to the Fermi level of the metal electrodes, and the coordination geometry at the metal-molecule interfaces. Recent investigations of QI effects present new opportunities for regulating charge transport through single-molecule junctions.^12–17^ This highlights the critical need to identify and assess novel approaches for the effective modulation of QI.^18^ Control of QI on the sub-molecular or molecular level would allow us to manipulate these phenomena with precision. These basic building blocks, comprising molecular switches that can controllably switch between “on” (high conductance) and “off” (low conductance) states, are vital towards developing molecular-scale computing, sensing, and memory devices. This feature is fundamental as switching mechanisms with high efficiency can lead to manipulation of signals in electronic systems at the molecular level.^19^ Consequently, different approaches have been designed that provide reversible and stable switching of the conductance of a single molecule between two or more distinguishable states. Studies have focused on exploring charge transport in conducting channels formed by π–π stacking conformations. The ability to manipulate the arrangement of the stacking^20–23^ structure adds a new feature of configurational control that is critical for the design and fabrication of QI-based supramolecular transistors. Other studies^24^ have shown that the manipulation of electrode connectivity to phenyl rings leads to switching QI patterns from constructive quantum interference (CQI) to destructive quantum interference (DQI). They show that bridging moieties across the biphenyl core effectively controlled.

**Fig. 1:**
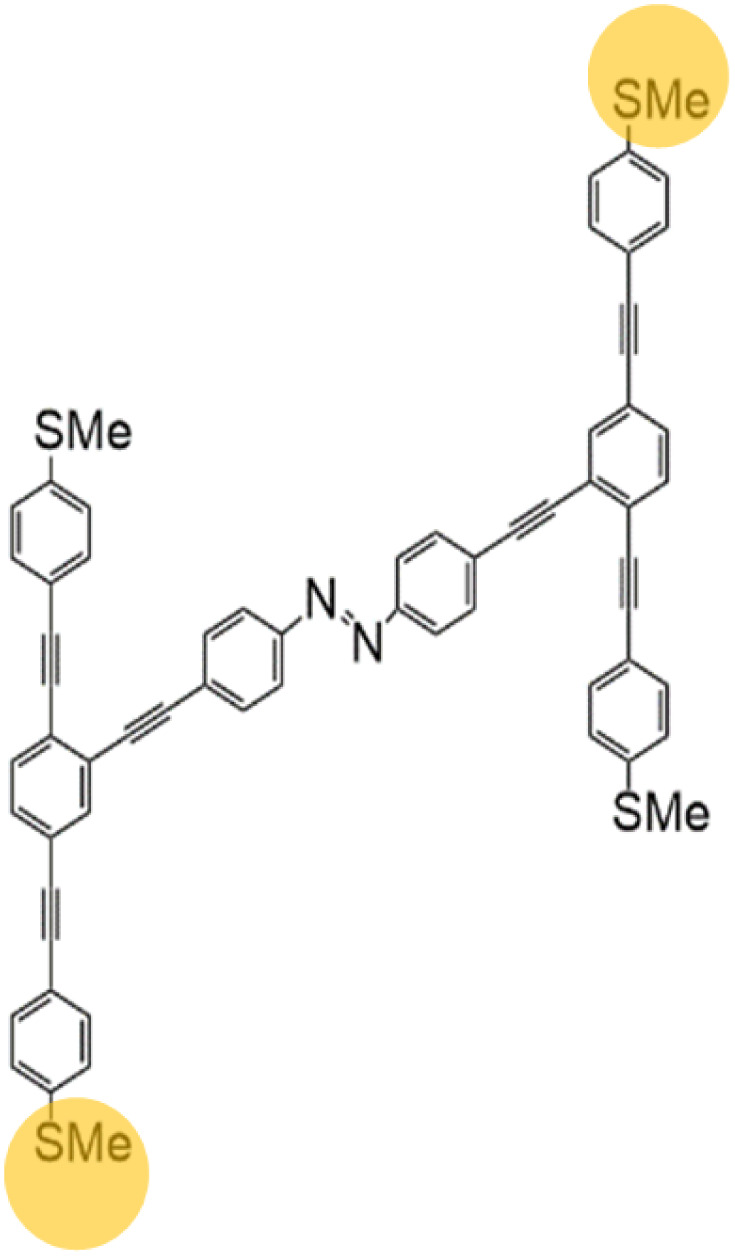
Schematic representation of an OPE3-based dimer, comprising of two OPE3 backbones linked *via* a conjugated azobenzene-containing bridge and terminated with thiomethyl (SMe) anchor groups. The molecule is designed to facilitate photoinduced isomerization about the azo double bond and quantum interference effects in single-molecule junctions.

In this work, we examine the switching ratio in OPE junctions^25^ formed from a 1,4-bis(phenylethynyl)benzene (OPE3) backbone, incorporating an azobenzene group as a bridging unit connecting two OPE3 backbone molecules, with thiomethyl (SMe) anchor groups, as schematically shown in Fig. 1. The OPE3-based molecule has three phenyl rings (benzene units) with ethylene (–C≡C–) linkages, forming a π-conjugated system.^26,27^ Azobenzene is a well-known functional group with two phenyl rings connected by an azo (–N=N–) bond, which serves as a key feature of its structure and functionality due to its *E*/*Z* photoisomerisation.^28,29^ The SMe groups are used as anchors to electrodes and consists of a sulfur atom attached to a methyl group (–CH_3_). It is commonly used as a substituent in organic compounds and is an essential component in organosulfur chemistry^30,31^ (for more detail see Fig. S2 and S3). Using the optimised structures, we employed the SIESTA code to compute self-consistent geometries, ground-state Hamiltonians, and overlap matrix elements for the studied molecules, as shown in Fig. S4. This study focuses on the electrical conductance of the OPE3-based dimer, specifically on configurations where only two anchoring groups are connected to metal electrodes, thereby enabling current to pass through the molecule. As illustrated in Fig. 1, two possible anchor pairs of connections are considered, as indicated by the yellow shading. This is similar to molecules examined in our previous study, shown in Fig. S1, which included a 1,4-diethynylbenzene group as the cross linking bridge between the two OPE3 backbones and showed switching from a high to low electrical conductance, with an on–off ratio of up to 3 orders of magnitude.^25^

**Fig. 2:**
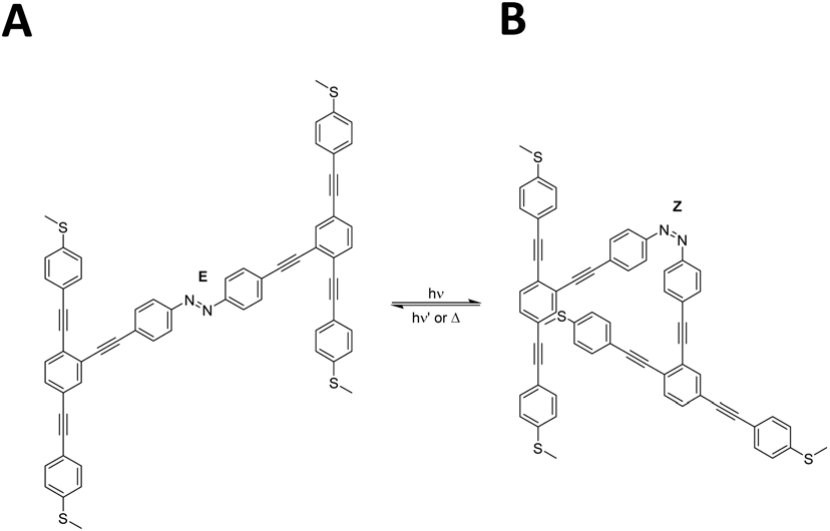
Schematic illustration of the OPE3-based molecule in an *E* or *Z* isomer under light stimulus, labelled A and B, respectively. A total energy difference of 0.04 eV is calculated between the fully relaxed geometries of the two isomers.

Light-induced conductance switching is an important topic in molecular electronics. Some commonly studied photochromic molecules include azobenzenes,^32–36^ dihydroazulenes^37^ and spiropyrans.^38^ Light has many advantages compared to other stimuli, such as remote application, fast response time, tuneable energy and non-invasive nature.^39^ When irradiated with light, the photoresponsive portion of these molecules typically undergoes either bond cleavage (open) or bond formation (closed), thereby reducing or enhancing conductance, respectively.^40–44^ The two distinct minimum energy values provide evidence that the molecule can stably exist in both configurations, suggesting the potential coexistence of these states under appropriate conditions^45–47^ as shown in Fig. 2.

## Methods

All theoretical simulations were conducted using the density functional theory (DFT) SIESTA code.^48^ The optimised geometries of the isomers of the OPE3-based molecule were obtained upon relaxing the isomers until the atomic forces became <0.01 eV Å^−1^ (see optimised DFT structures of isolated configurations in Fig. S4, SI) We employed a double zeta plus polarization orbital basis set, norm-conserving pseudopotentials, and local density approximation GGA exchange–correlation functional, with a real space grid defined by an energy cut-off of 250 Rydberg. Furthermore, we performed calculations while using the generalized gradient approximation GGA, obtaining very similar structures as in the LDA case.^49–51^

## Results and discussion

Upon light irradiation, the OPE3-based molecule isomerises from an *E* to *Z* configuration, referred to A and B, respectively, with their structures shown in Fig. 2. Fully relaxed configurations of both isomers were obtained by allowing the systems to reach their minimum energy geometries, as shown in Fig. S9. To confirm their relative stability, we computed the total energies of both structures, enabling a comparative analysis of their energetics. Our DFT simulations demonstrated that both structures are stable, with a total energy difference of only 0.04 eV between the two isomers.

**Fig. 3:**
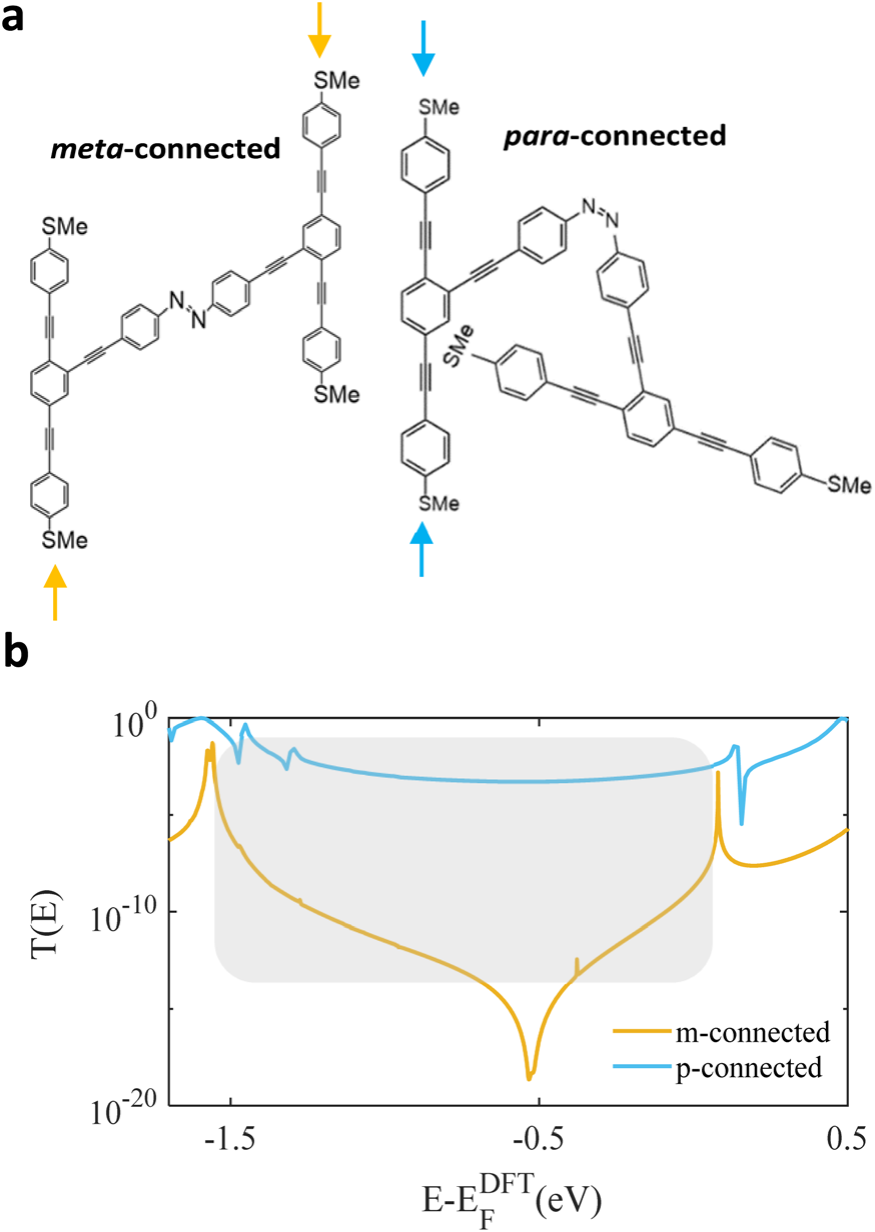
(a) Schematic illustration of the two OPE3-based structures *meta*- and *para*-connected (*E* and *Z* on the left and right). Dark orange arrows indicate *meta*-connection, while the light blue is *para*-connection. (b) Transmission coefficient *T*(*E*) of both junctions against electron energy *E*. Shaded area shows the comparison area.

In a study by Jia *et al.*,^52^ the authors investigated a single-molecule junction applied with a photoactive diarylethene unit designed with three methylene groups to generate weak connections to graphene electrodes. Upon exposure to UV light, the diarylethene molecule switches from an open conformation, which has low conductance (off state), to a closed conformation with high conductance (on state). Conversely, it returns to the open state under exposure to visible light. In this current study, we investigate the electrical properties of an OPE3-based structure incorporating an azobenzene bridge, aiming to evaluate switching characteristics and potential for use in molecular electronic devices. Fig. 3(a) shows the two isomers of the OPE3-based molecule with arrows showing the thiomethyl groups the electrodes are connected to, which are either *meta*- (left) or *para*-connected (right) due to the azobenzene photoisomerization. We calculated the transport properties with two different pairs of contacts to electrodes for each of the two isomers. The HOMO, LUMO, and their neighbours (*i.e.*, HOMO+1, LUMO+1 *etc.*), along with their energies are shown in Fig. S5 and S6 (SI). Fig. 3(b) shows the transmission coefficient of the *meta*-connected *E* isomer (orange) switching from a destructive QI to the constrictive *para*-connected *Z* isomer (light blue). The photoisomerization of the azobenzene group, results in the rotation of the second OPE3 backbone.^53–55^ These reversible changes in azo-bonding are ideal for the conductance switching behaviour between on and off states. We investigated the role of connectivity in leading to high and low electrical conductivity associated with CQI and DQI.^56^ In particular, we found that DQI emerges when the transport path contains one or more *meta*-connections. The photochromic azobenzene allows the molecule to be isomerized between on or off states, with an on/off transmission ratio of between 5 and 10 orders of magnitude over a wide range of energy >1.5 eV, as indicated by the shaded region in Fig. 3b. This demonstrates that light-induced switching can produce on–off ratios that are significantly larger than published ratios for similar molecules.^14,57–60^ This large ratio arises, because transport through the left junction in Fig. 3a takes place *via* a *meta*-connected phenyl ring in the right OPE3 backbone, leading to DQI. Whereas transport through the right junction in Fig. 3b takes place *via* a *para*-connected phenyl ring, leading to CQI.

To realise the above switching experimentally, we envisage starting from the *E* isomer in the “off” position, bound to the substrate by the lower SMe group. As an STM or conductive AFM tip approaches the *E* isomer from above, it will first encounter the SMe group at the position of the yellow arrow and therefore measure the low “off” conductance. After withdrawing the tip, shining light onto the trans molecule causes it to flip to the *Z* isomer (Fig. 3a, left to right). As an STM or conductive AFM tip approaches the *Z* isomer from above, with a high probability, it will first encounter the SMe group at the position of the blue arrow and therefore measure the higher *Z* conductance.

**Fig. 4:**
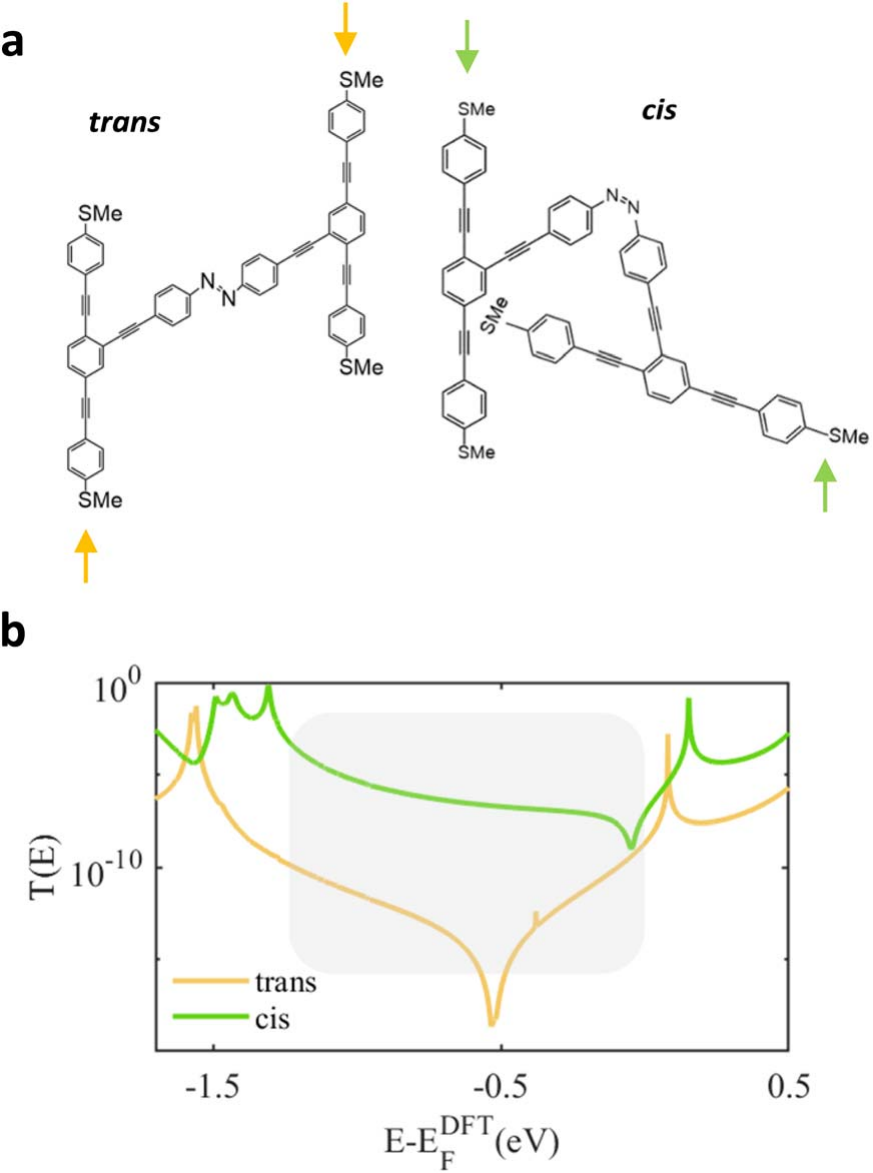
(a) Schematic illustration of the two OPE3-based structures with *E* and *Z* conformations. (b) Transmission coefficient *T*(*E*) of both junctions against electron energy *E*, obtained when electrodes are connected to the SMe groups indicated by arrows.

In real scanning tunneling microscopy (STM) break-junction experiments, in the *Z* form, it may be possible to connect to the molecule at the points indicated by the green arrows in Fig. 4a, whose resulting transmission coefficient is shown in Fig. 4b. Within the HOMO–LUMO gap (indicated by the shaded area) the cis transmission coefficient is again significantly greater than the that of the trans molecule and therefore a large conductance ratio is still expected when *E*–*Z* isomerisation is induced.

## Conclusion

We have demonstrated that photoisomerization of the azobenzene linker in an OPE3-based dimer can cause the electron transport to switch from DQI, (corresponding to a low-conductance off state) to CQI (corresponding to a high-conductance on state). In the off state, the transmission coefficient *T*(*E*) is between 5 and 10 orders of magnitude smaller than that of the on state over a large energy range within the HOMO–LUMO gap, and therefore the electrical conductance 
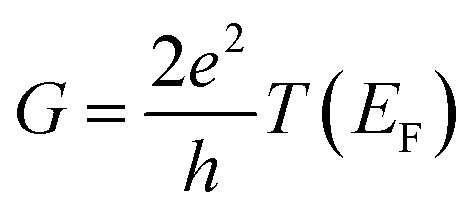
 is between 5 and 10 orders of magnitude smaller than that of the on state, depending on the precise value of the Fermi energy *E*_F_. This large on–off conductance ratio opens new avenues for creating molecular-scale devices, with favourable switching characteristics.

## Author contributions

The manuscript was written through contributions of all authors. All authors have given approval to the final version of the manuscript. A. K. I., conceived the research. A. A., B. A., K. A. and K. A. carried out the simulations. All co-authors assisted in writing the manuscript. A. K. I. and C. J. L. supervised the research and provided essential contributions to interpreting the results and drafting the manuscript.

## Conflicts of interest

There are no conflicts to declare.

## Supplementary Material

NA-OLF-D5NA00553A-s001

## Data Availability

In this work, we use the following codes: 1 – Siesta code used to predict the Hamiltonian of each system used in this study, which is located in https://gitlab.com/siesta-project/siesta/-/releases; 2 – GOLLUM software is used to find the website's transmission coefficient. https://www.gollumcode.com/; 3 – conductance, Seebeck, and other parameters are calculated using the own Fortran code available upon request. Supplementary information is available. See DOI: https://doi.org/10.1039/d5na00553a.
