# Supplementary material for: Light-driven molecular switching achieves 6-order magnitude conductance change in OPE dimers
Source: Nanoscale Adv. 2025 Oct 24;7(24):7958–63. doi: 10.1039/d5na00553a (PMC12551148; doi:10.1039/d5na00553a)
Supplement: NA-007-D5NA00553A-s001 [file NA-007-D5NA00553A-s001.pdf]

Supplementary Information (SI) for Nanoscale Advances.
This journal is © The Royal Society of Chemistry 2025

Light-driven molecular switching achieves 6-order magnitude conductance change in OPE Dimers

Asma Alajmi,[a†] Bashayer Alanazi,[a†] Karimah Alresheedi,[a†] Kholood Alharbi,[a†] William D. J. Tremlett,[b] Nicholas J. Long,[b] Colin Lambert[a*] and Ali Ismael[a*]

[a.]Physics Department, Lancaster University, Lancaster, LA1 4YB, UK.
[b] Department of Chemistry, Imperial College London, London W12 0BZ, UK.
† These authors contributed equally to this work
* c.lambert@lancaster.ac.uk , and Ali Ismael: k.ismael@lancaster.ac.uk

Contents

1. **Interdiction** 2
2. **Optimised DFT structures of isolated molecular-scale structures.** 4
3. **Frontier molecular orbitals** 5
   3.1 Cross-linked (A) 5
   3.2 Cross-linked (B) 7
4. **Orbital product rule (OPR)** 8
5. **Binding energies** 9
6. **Geometries of molecules and angles between the two states dimer A to dimer B** 11
7. **Transport simulation in Au-Au junction** 12
8. **Comparison of study results** 14
9. **References** 16

1. Interdiction

In our previous paper [1] *"Tuning Quantum Interference through Molecular Junctions Formed from Cross-Linked OPE-3 Dimers,"* we investigated the switching ratio associated with the connectivity points within the molecular system [1]. To investigate the transition from constructive to destructive quantum interference, we studied two OPE3-based dimers incorporating different bridging units, as illustrated in **Figure (S. 1)**. These molecular structures adopt a staggered configuration, resulting in six distinct connectivity sites.

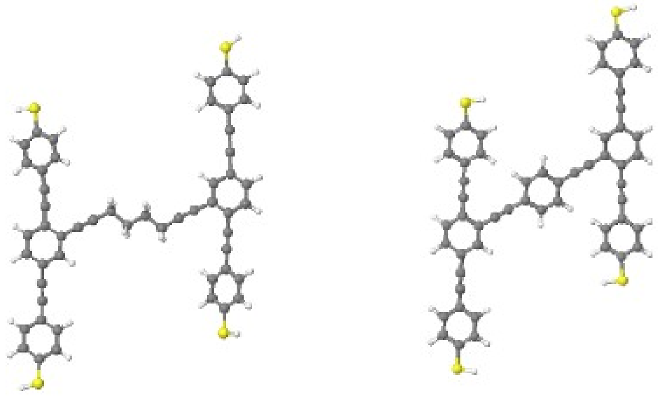

Figure (S. 1): Schematic representations of two cross-linked dimers, featuring six connecting points, were used to determine the switching ratio [1].

In this current study, we investigate a new bridging unit *azobenzene* known for its distinctive photoresponsive properties. We focus on evaluating the switching ratio associated with the photoisomerization of the azo (N=N) bond within the azobenzene moiety. The chemical structure of the azobenzene unit is shown in **Figure (S. 2)** and will be discussed in detail throughout this work.

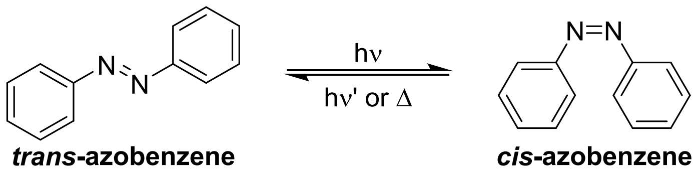

The molecule studied features two 1,4-bis(phenylethynyl)benzene (OPE3) backbone units bridged by an azobenzene group to create an OPE3-based dimer, with thiomethyl (SMe) groups serving as the anchors to the electrodes, shown in **Figure (S. 3)**. OPE3 is a conjugated organic molecule that combines three phenyl rings (benzene units) and two ethylene ($-C \equiv C-$) linkages, forming a π-conjugated system[2, 3]. Azobenzene is a well-known organic functional group consisting of two phenyl rings connected by an azo ($-N=N-$) bond, which serves as a key feature of its structure and functionality due to its *E/Z* (*trans/cis*) photoisomerisation [4, 5]. The SMe groups were employed as anchors to electrodes and comprises of a sulfur atom bonded to a methyl group ($-CH_3$). It is frequently used as a substituent in organic compounds and plays a significant role in various chemical reactions, particularly within the field of organosulfur chemistry [6, 7].

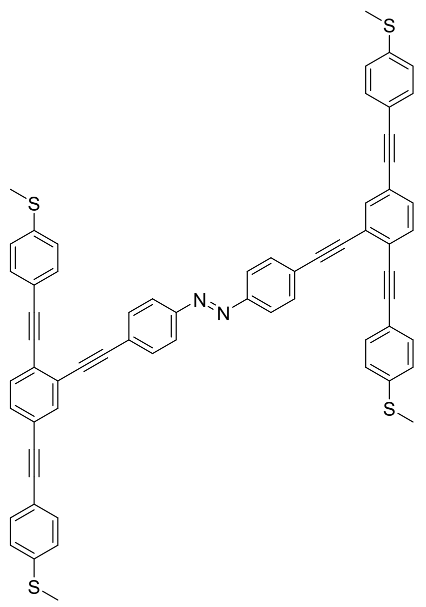

2. Optimised Density Functional Theory (DFT) structures of isolated molecular-scale structures.

The SIESTA code was used to calculate the optimised geometries. The molecule depicted in **Figure (S. 3)** was optimised by relaxing the structure to obtain the most stable geometry, which happens when all forces are less than $0.01 eV$. A real-space grid is defined by a double-polarized basis set (DZP) with a $250\ Ry$ equivalent energy cutoff. The generalised gradient approximation (GGA) is selected to be the exchange-correlation functional. **Figure (S. 4)** shows fully relaxed geometries of the studied molecule in two isomeric forms. Structure A shows the molecule with the azobenzene group in an *E* (*trans*) conformation. Upon exposure to light, the azobenzene group undergoes an *E* to *Z* isomerisation, resulting in the formation of structure B as shown in **Figure (S. 4)** [8-10]. To identify specific connectivity configurations conducive to high conductance and those leading to lower levels of electrical conductivity (CQI and DQI[11]).

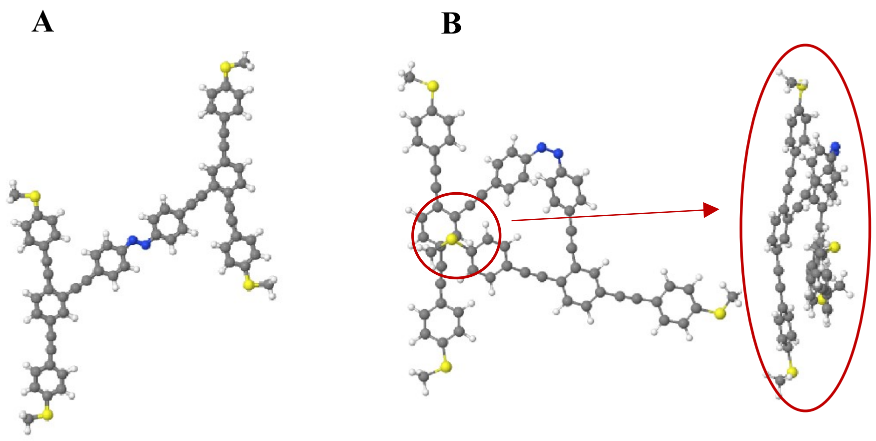

Figure (S. 4): Fully relaxed calculated molecular structures of the OPE3-based molecule as either *E* (**A**) or *Z* (**B**) isomers.

3. Frontier molecular orbitals

To have a better understanding of the electronic properties of the isomers in **Figure (S. 4)**, the gas-phase electronic structures were investigated to explore the distribution and composition of the frontier molecular orbitals. Plots of the frontier orbitals for the *E* and *Z* isomers of the OPE3-based dimer are given in **Figure (S. 5)** and **Figure (S. 6)**, respectively, which show the highest occupied molecular orbitals (HOMO), lowest unoccupied orbitals (LUMO), (HOMO-1), (LUMO+1), (HOMO-2) and (LUMO+2) along with their energies. The blue and red colours represent the positive and negative orbital amplitudes, respectively.

3.1 Cross-linked (A)

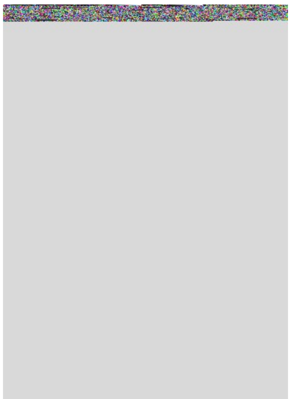

E_F= -3.56 eV

a

HOMO= - 4.20 eV

LUMO= - 2.89 eV

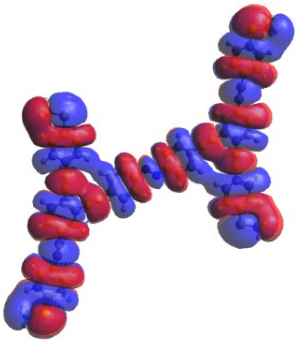

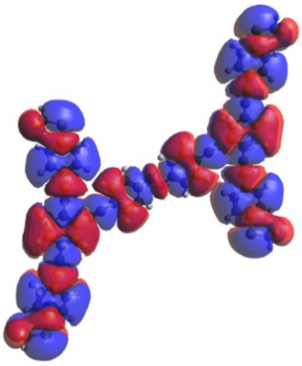

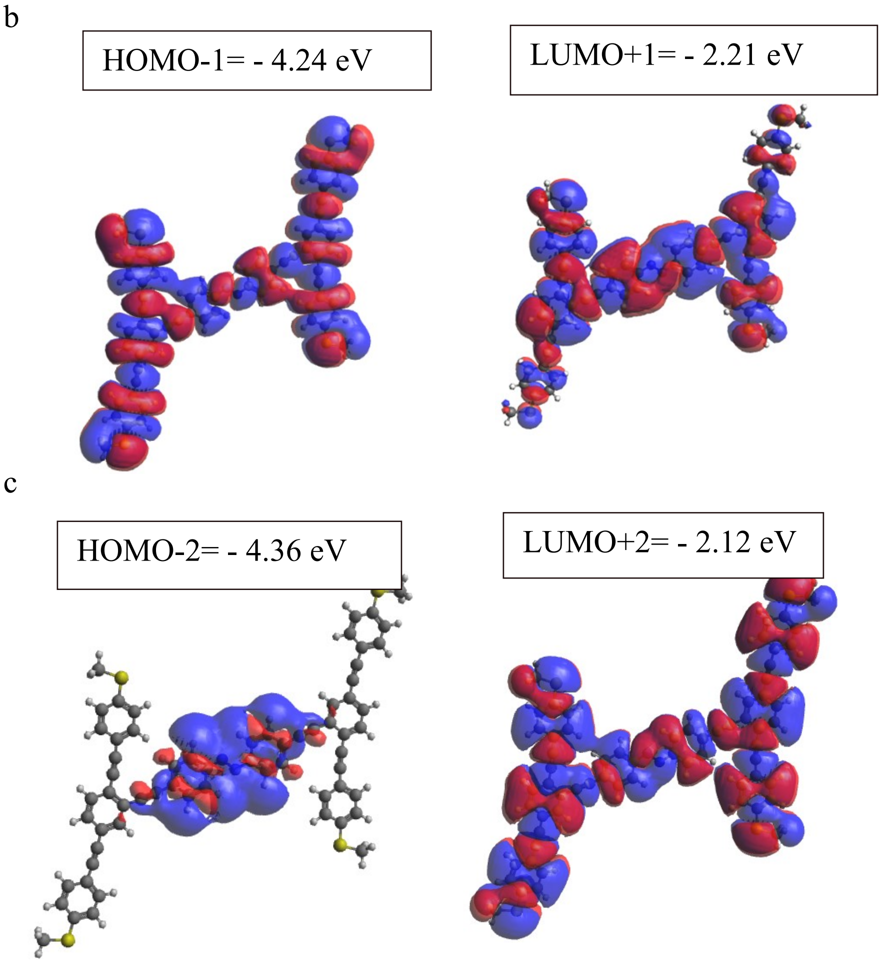

Figure (S. 5): Wave function for isomer **A**. **Top panel:** fully optimised geometry. Lower panel: HOMO, LUMO, HOMO-1, LUMO+1, HOMO-2 and LUMO+2 of isomer **A**, along with their energies.

3.2 Cross-linked (B)

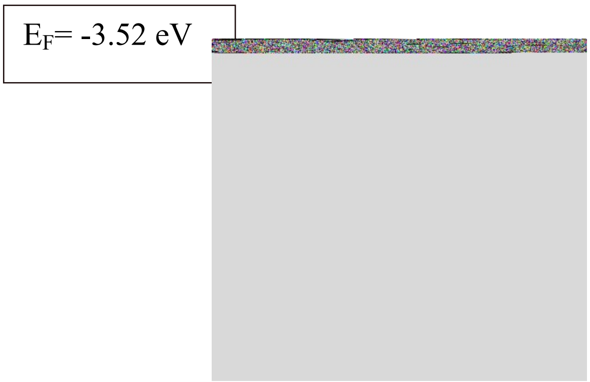

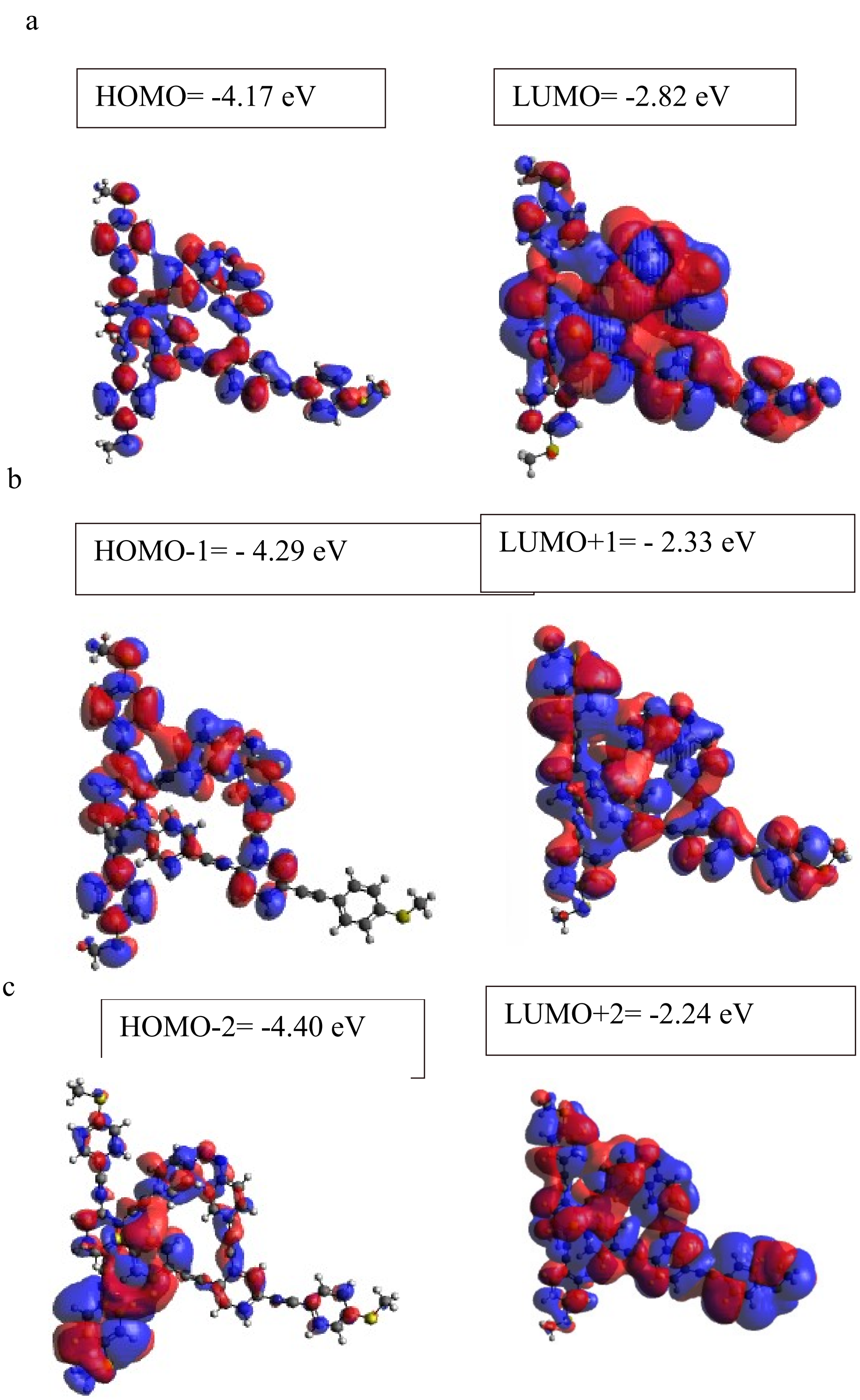

Figure (S. 6): Wave function for isomer **B**. **Top panel:** fully optimised geometry. Lower panel: HOMO, LUMO, HOMO-1, LUMO+1, HOMO-2, LUMO+2 of isomer **B**, along with their energies.

4. Orbital product rule (OPR)

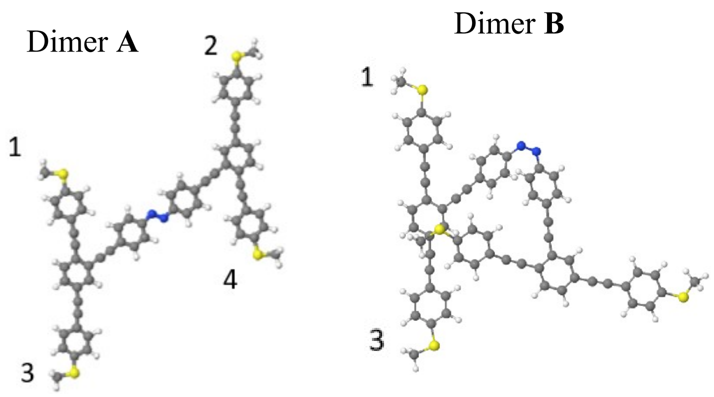

Figure (S. 7): Schematic illustration of isomer **A** with two possible contact points (positions *2* and *3*), and isomer **B** with contact points at positions *1* and *3*.

The orbital product rule (OPR) is a concept used to predict quantum interference effects in molecular systems, particularly in molecular electronics. A deeper understanding of the relationship between molecular orbitals and electron transport through a molecule can be obtained by evaluating whether the orbitals' phases contribute to CQI or DQI quantum interference [12, 13]. It is worth noting that the studied isomers exhibit different potential contact points: for isomer **A** the connectivity occurs at positions *2* and *3*, whereas for isomer **B** the contact points are located at positions *1* and *3*, as shown in **Figure (S. 7)**.

Table S1 illustrates the OPR predictions for the studied isomers **A** and **B**. The product rule applies on the contact points to determine whether the quantum interference is constructive or distructive. The OPR predicts a DQI for contact points at position *2* and *3* corresponding to isomer **A**, whereas the OPR predicts a CQI for contact points *1* and *3* for isomer **B**, and their predictions are presented in Table S1.

Table 1: Orbital Product Rule predictions of contact points *2 + 3* and *1 + 3*, for isomers **A** and **B**, respectively. (Note: C for constructive and D for destructive QI).

Isomer	Contact points	OPR prediction	Wavefunction plot Figure
**A**	*2-3*	D	Figure (S. 4a)
**B**	*1-3*	C	Figure (S. 5a)

5. Binding energies

We need to attach the discrete dimers to two metal electrodes so that we can calculate the conductance. In addition, we need to determine the distance between the isolated molecule and electrodes. To calculate the distance between two objects we have to find the binding energy which is defined as the optimum distance between two objects. The binding energy is calculated by using the DFT method which is required to calculate the ground state energy of the total system. Using DFT code like SIESTA are subject to errors. A major problem with this kind of calculation is the interaction energies in closing atoms due to the overlap of their basic functions which affects the total energy of the system. These errors can be removed by applying the counterpoise correction. A possible explanation for this is calculating the energy in the same total basis set AB by removing the numerical errors using the 'ghost' states (basis set functions which have no electrons or protons) in SIESTA. Thus, the total energy of the systems A and B in the isomer basis can be expressed by the following equation:

$$Binding\ Energy = E_{AB}^{AB} - E_{A}^{AB} - E_{B}^{AB} \qquad (1)$$

In this section, the optimal distances between the Au electrode and various molecules were determined, as illustrated in **Figure (S. 8)**:

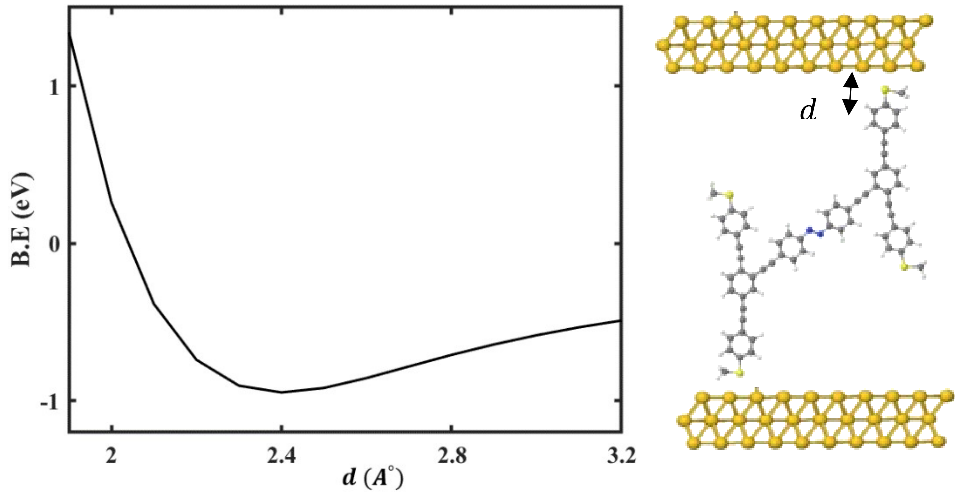

Figure (S. 8): Right panel: represents OPE3-based molecule binding to a gold atom. **Left panel:** Binding energy as a function of the optimum binding distance , where is found to be approximately , and binding energy .

6. Geometries of molecules and angles between the two states dimer A to dimer B

The DFT code (SIESTA) was employed to obtain fully relaxed geometries of the isomers **A** to **B**, as well as their torsion angles (see **Figure (S. 9)**). It is well known that DFT is not able to distinguish between the local minima and global minima, which means the relaxation stops with the first total minima energy whether it is local or global [14]. To find the global minima we fixed one of the OPE3 backbone and rotated the other 96° around the Z-axis, as shown in **Figure (S. 9)**, and at each angle of rotation we calculated the total energy. After finding the global minima (the lowest energy), we allowed the molecules to fully relax around the global minima angles so that we can obtain the right angle of the molecules. Our DFT simulations demonstrated that both structures are stable, with a total energy difference of only 0.04 eV.

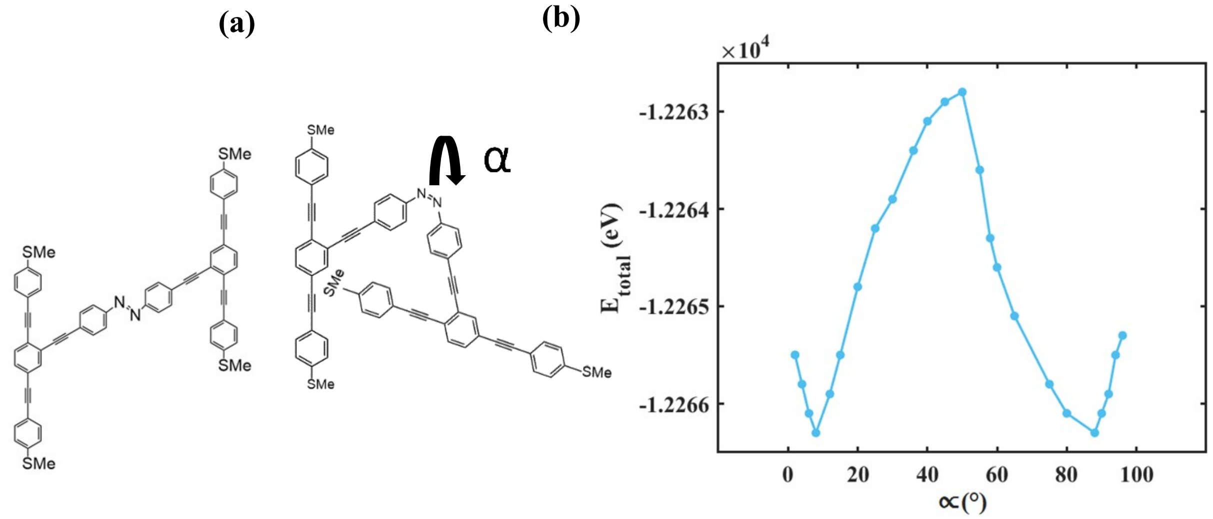

Figure (S. 9): (a) Fully optimised geometry of OPE3-based isomers (A and B), with the right angle. **(b)** The global minima energy for isomers **A** and **B**.

7. Transport simulation in Au-Au junction

In the following transport calculations, the ground state Hamiltonian and optimised geometry of each compound were obtained using the DFT code. The generalized gradient approximation (GGA) exchange-correlation functional was used along with double zeta polarised (DZP) basis sets and the norm-conserving pseudopotentials. The real space grid was defined by a plane wave cut-off of $250\ Ry$ [15, 16]. The geometry optimisation was carried out to a force tolerance of $0.01\ eV/Å$. This process was repeated for a unit cell with the molecule between two electrodes where the optimised distance between electrodes and the anchor groups is shown in **Figure (S. 10)** and **Figure (S. 11)**. From the ground state Hamiltonian, the transmission coefficient, and the room temperature electrical conductance G was obtained, as described in the sections below. In this section, we shall calculate the transport in gold junctions as follows:

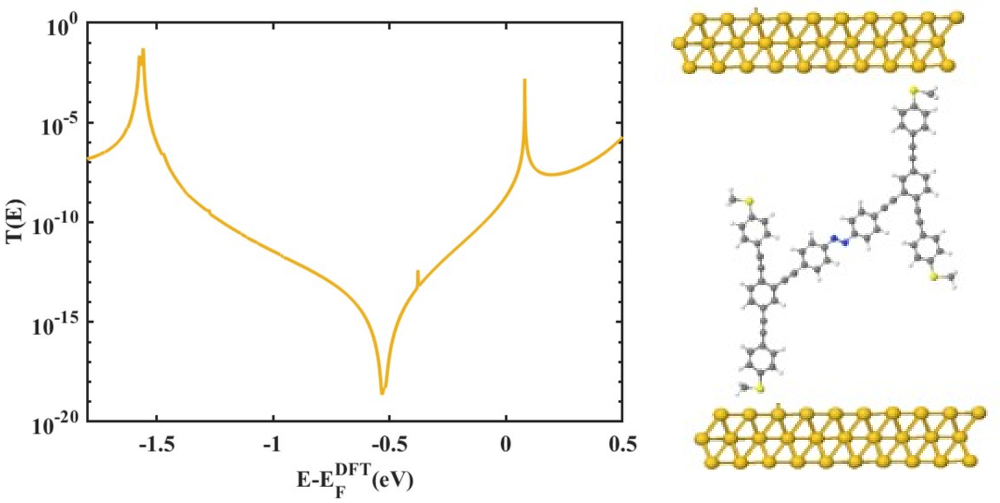

Figure (S. 10): Right panel: Schematic illustration of the Au/**A**/Au junctions in **2** + **3** contact points. **Left panel:** Transmission coefficient of Au/**A**/Au junctions against electron energy .

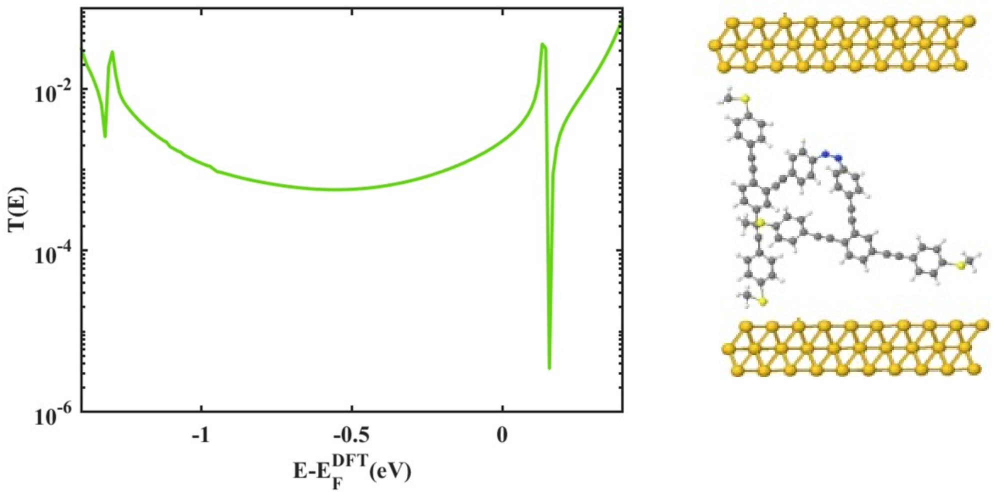

Figure (S. 11): Right panel: Schematic illustration of the Au/**B**/Au junctions in **1** + **3** contact points. **Left panel:** Transmission coefficient of Au/**B**/Au junctions against electron energy .

Table 2: Orbital product rule predictions for isomers **A** and **B**, against the DFT and TBM results (Note: ✓ = OPR and DFT agree while x = OPR and DFT don't agree).

Isomer	Contact points	OPR prediction	DFT	OPR and DFT agreement	Wavefunction plot Figures
**A**	*2-3*	C	C	✓	**Figure (S. 4) (a)**
**B**	*1-3*	D	D	✓	**Figure (S. 5) (a)**

8. Comparison of study results

The photochromic side group of azobenzene facilitates reversible isomerization between two distinct states, commonly referred to as the *on* and *off* configurations. Our measurements reveal a reversible switching behavior in the molecular conductance, characterized by an on/off conductance ratio of approximately 6. Notably, the transition from constructive quantum interference (CQI) to destructive quantum interference (DQI) is consistently observed irrespective of the chemical nature of the bridging linker. This transition yields an on/off conductance ratio spanning up to six orders of magnitude, significantly surpassing the three orders of magnitude reported in previous studies.

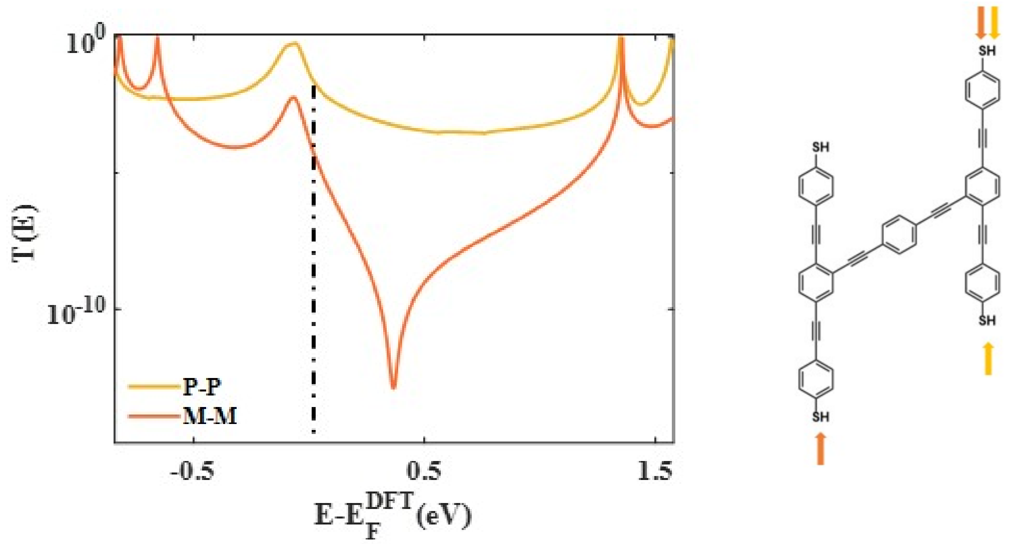

Figure (S. 12): Right panel: Schematic illustration of the cross-linked junctions in two different contact points. **Left panel:** Transmission coefficient of cross-linked junctions against electron energy .

(a) **(b)**

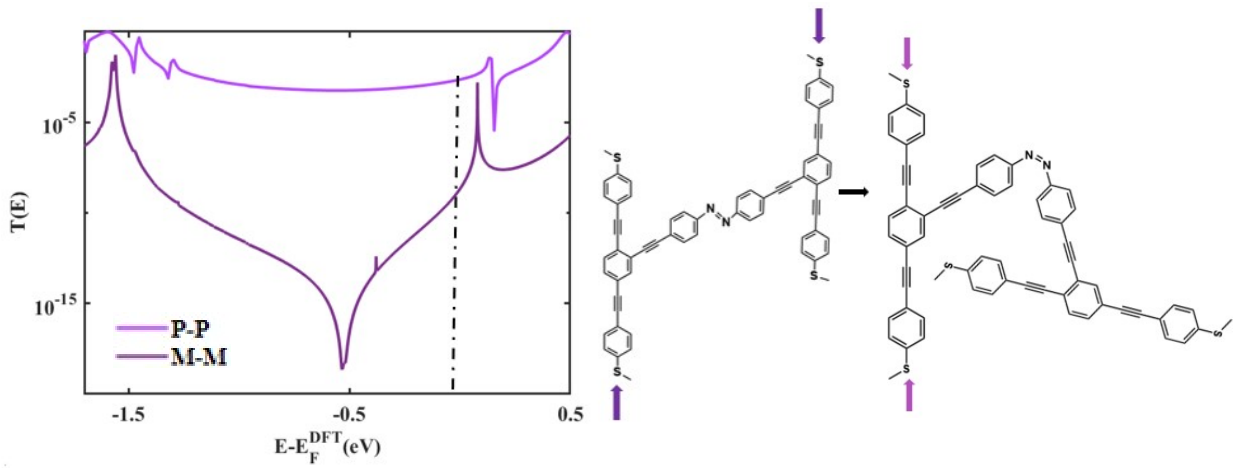

Figure (S. 13): Right panel: Schematic illustration of the Au/**A**/Au and Au/**B**/Au junctions in two different contact points. **Left panel:** Transmission coefficient of Au/**A**/Au and Au/**B**/Au junctions against electron energy .

9. References

[1] B. Alanazi, A. Alajmi, A. Aljobory, C. Lambert, A. Ismael, Tuning quantum interference through molecular junctions formed from cross-linked OPE-3 dimers, J Mater Chem C, 12 (2024) 6905-6910.
[2] T. Ryhding, M.-B.S. Kirketerp, U. Kadhane, M.K. Lykkegaard, S. Panja, S.B. Nielsen, M.B. Nielsen, Upon the intrinsic optical properties of oligo (p-phenyleneethynylene) s (OPEs). Synthesis of OPE3 for experimental gas-phase absorption studies, Tetrahedron, 64 (2008) 11475-11479.
[3] A. Gopal, R. Varghese, A. Ajayaghosh, Oligo (p-phenylene-ethynylene)-Derived Super-π-Gelators with Tunable Emission and Self-Assembled Polymorphic Structures, Chemistry–An Asian Journal, 7 (2012) 2061-2067.
[4] C.t. Brown, A refinement of the crystal structure of azobenzene, Acta Crystallographica, 21 (1966) 146-152.
[5] J. Bouwstra, A. Schouten, J. Kroon, Structural studies of the system trans-azobenzene/trans-stilbene. I. A reinvestigation of the disorder in the crystal structure of trans-azobenzene, C12H10N2, Acta Crystallographica Section C: Crystal Structure Communications, 39 (1983) 1121-1123.
[6] C. He, Y. Wang, Z. Li, Y. Huang, Y. Liao, D. Xia, S. Lee, Facet engineered α-MnO_2 for efficient catalytic ozonation of odor CH_3SH: oxygen vacancy-induced active centers and catalytic mechanism, Environmental science and technology, 54 (2020) 12771-12783.
[7] Y. Ouyang, Effect of zinc on the metabolism of thiol-treated human gingival fibroblasts, in, University of British Columbia, 1991.
[8] H. Kandori, Y. Shichida, T. Yoshizawa, Photoisomerization in rhodopsin, Biochemistry (Moscow), 66 (2001) 1197-1209.
[9] R. Liu, A.E. Asato, The primary process of vision and the structure of bathorhodopsin: a mechanism for photoisomerization of polyenes, Proceedings of the National Academy of Sciences, 82 (1985) 259-263.
[10] F. Gallego-Gómez, F. Del Monte, K. Meerholz, Optical gain by a simple photoisomerization process, Nature materials, 7 (2008) 490-497.
[11] M. Prauzek, J. Konecny, M. Borova, K. Janosova, J. Hlavica, P. Musilek, Energy harvesting sources, storage devices and system topologies for environmental wireless sensor networks: A review, Sensors, 18 (2018) 2446.
[12] C.J. Lambert, S.X. Liu, A magic ratio rule for beginners: a chemist's guide to quantum interference in molecules, Chemistry–A European Journal, 24 (2018) 4193-4201.
[13] M. Famili, C. Jia, X. Liu, P. Wang, I.M. Grace, J. Guo, Y. Liu, Z. Feng, Y. Wang, Z. Zhao, Self-assembled molecular-electronic films controlled by room temperature quantum interference, Chem, 5 (2019) 474-484.
[14] B. de Souza, GOAT: A Global Optimization Algorithm for Molecules and Atomic Clusters, Angewandte Chemie International Edition, (2025) e202500393.
[15] E. Artacho, E. Anglada, O. Dieguez, J.D. Gale, A. Garcia, J. Junquera, R.M. Martin, P. Ordejon, J.M. Pruneda, D. Sanchez-Portal, J.M. Soler, The SIESTA method; developments and applicability, J Phys Condens Matter, 20 (2008) 064208.
[16] L.A. Zotti, T. Kirchner, J.C. Cuevas, F. Pauly, T. Huhn, E. Scheer, A. Erbe, Revealing the role of anchoring groups in the electrical conduction through single-molecule junctions, small, 6 (2010) 1529-1535.
